# Comparison of obesity-related indices for identifying nonalcoholic fatty liver disease: a population-based cross-sectional study in China

**DOI:** 10.1186/s12944-021-01560-3

**Published:** 2021-10-10

**Authors:** Fangfei Xie, Yuyu Pei, Quan Zhou, Deli Cao, Yun Wang

**Affiliations:** Physical Examination Center, the Affiliated Suzhou Hospital of Nanjing Medical University, Suzhou, Jiangsu People’s Republic of China

**Keywords:** Nonalcoholic fatty liver disease, Obesity, Indices, Anthropometric, Metabolic, Prediction, Diagnostic ability

## Abstract

**Background:**

The relationship between nonalcoholic fatty liver disease (NAFLD) and obesity-related indices has been analyzed separately thus far, and evidence comparing these indices together is still lacking, especially in China. This study aimed to comprehensively evaluate the predictive performance of anthropometric and metabolic indices to identify NAFLD in Chinese adults.

**Methods:**

This study recruited a total of 1748 participants who were 18 years or older in southeastern China. The systolic blood pressure (SBP), diastolic blood pressure (DBP), fasting blood glucose (FBG), total cholesterol (TC), triglycerides (TGs), low-density lipoprotein (LDL), waist circumference (WC), a body shape index (ABSI), atherogenic index of plasma (AIP), abdominal volume index (AVI), body adiposity index (BAI), body mass index (BMI), body roundness index (BRI), conicity index (CI), triglyceride glucose (TyG), waist hip ratio (WHR), and waist height ratio (WHtR) were measured. The association between these indices and NAFLD was analyzed via logistic analyses with odds ratios (ORs). Receiver operating characteristic (ROC) curves and areas under the curve (AUCs) were used to compare the predictive performance of these indices to identify NAFLD.

**Results:**

BMI had the greatest total AUC (AUC = 0.841) in the ROC curve analysis. However, BRI and BMI both had the best diagnostic ability in males (AUC = 0.812), and BRI had the best diagnostic ability in females (AUC = 0.849). Furthermore, AVI had the greatest AUC for patients who were ~ 20 (AUC = 0.892) and ~ 40 years old (AUC = 0.831), while TyG showed a higher predictive ability than AVI in those who were ~ 60 years old (AUC = 0.766).

**Conclusion:**

This study identified sex- and age-specific indices for predicting NAFLD in Chinese subjects. Compared with indices for all age groups, sex- and age-specific indices can provide more accurate assistance for clinical diagnosis and treatment.

**Supplementary Information:**

The online version contains supplementary material available at 10.1186/s12944-021-01560-3.

## Background

It is well established that nonalcoholic fatty liver disease (NAFLD) has become a major public health problem over the past few decades, with incidences of approximately 30 and 25% in Western and Asian countries, respectively. NAFLD has also caused large medical and economic burdens for both developed and developing countries [1–5]. NAFLD is also characterized by complex pathogenesis and is difficult to diagnose [6, 7]. Thus, it is of great necessity to further explore the pathogenesis of NAFLD to determine effective predictive indicators for the diagnosis of NAFLD, which is critical for the prevention and treatment of NAFLD.

Oxidative stress and inflammation can promote NAFLD to nonalcoholic steatohepatitis (NASH) or even hepatic cirrhosis in the progression of NAFLD [8]. Although the pathogenesis of NAFLD is still not fully understood, obesity has been demonstrated to play a major role in most of the pathogenic pathways involved in NAFLD. Dietary nutrients have played an increasingly important role in the progression of NAFLD in recent years by affecting lipid and carbohydrate metabolism. For example, an obesogenic diet is associated with hepatic oxidative stress and inflammation, which might be due to the activation of anabolic pathways and can eventually lead to abdominal obesity [9]. In contrast, the intake of polyunsaturated fatty acids (n-3 polyunsaturated fatty acids) can reduce nutritional hepatic steatosis in adults, which favors fatty acid and TG production over fatty acid oxidation [10].

NAFLD is commonly associated with visceral adiposity, type II diabetes, dyslipidemia, and metabolic disorders [11–15]. The relationship between NAFLD and type II diabetes is complex and bidirectional and occurs in the context of a broader association between NAFLD and metabolic syndrome [16, 17]. In recent decades, the prevalence of NAFLD has increased alarmingly, along with increasing rates of obesity [18]. Some studies found that the development of NAFLD may be influenced by the regional distribution of lean and fat mass and suggested that abdominal fat is a risk factor for both fatty liver and fatty liver fibrosis [7, 14]. Therefore, obesity-associated factors might be utilized for predicting NAFLD.

As expected, several anthropometric or metabolic indices, such as the atherogenic index [19, 20], body mass index (BMI) [21], triglycerides (TGs)/high-density lipoprotein cholesterol [22], visceral adipose tissue [23], total cholesterol (TC)/high-density lipoprotein cholesterol [24], triglyceride glucose index (TyG) [25, 26], and blood pressure [27] have been reported to be associated with NAFLD in both cross-sectional and cohort studies. However, most existing studies mainly focused on only one or two indices, which might have limitations for predicting NAFLD considering the high complexity of the pathogenesis of NAFLD. Meanwhile, it remains unclear which indices might be even more advantageous than others for predicting NAFLD. This is especially true for Chinese subjects since the prevalence of overweight and obesity has increased considerably in China [28]. This study aimed to evaluate the performance of obesity-related indices, including the systolic blood pressure (SBP), diastolic blood pressure (DBP), fasting blood glucose (FBG), TC, TGs, low-density lipoprotein (LDL), waist circumference (WC), body shape index (ABSI), atherogenic index of plasma (AIP), abdominal volume index (AVI), body adiposity index (BAI), BMI, body roundness index (BRI), specificity index (CI), TyG, waist hip ratio (WHR) and waist height ratio (WHtR), in identifying NAFLD in Chinese adults. Hopefully, the result will provide a theoretical basis for utilizing anthropometric and metabolic indices to predict NAFLD in China.

## Methods

### Study population

**Fig. 1:**
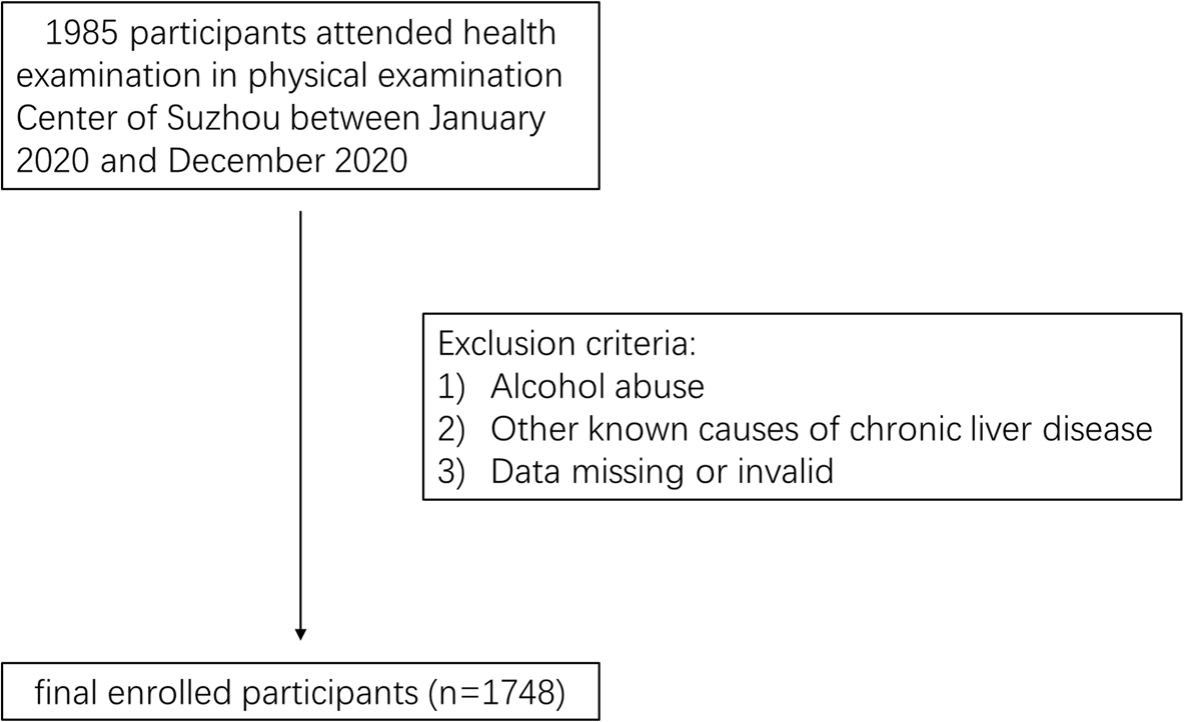
Flow chat

Participants were recruited in the physical examination center of Suzhou in southeastern China from January 2020 to December 2020 cross-sectionally. Subjects included in this study were of Chinese Han ethnicity and were over 18 years old. A total of 1748 subjects were finally included in the analysis after excluding those with alcohol abuse, other known causes of chronic liver disease and missing or invalid data (Fig. 1). The ethical committee of the Affiliated Suzhou Hospital of Nanjing Medical University approved this study (approval no. KL9011 Study 12), and the study also obtained approval from all subjects who had agreed to participate in the present study.

### Data collection

The morning health examination was performed by expert medical staff. Health checkups and blood markers were measured as described previously by Xie et al. [19]. In brief, weight and height were measured in light indoor clothing without shoes and heavy clothes using a calibrated measuring apparatus. WC and hip circumference (HC) were measured as the horizontal circumference that passes through the navel position and the bulge at the hip, respectively. SBP and DBP were measured by sphygmomanometer. Metabolic markers, including TC, TG, LDL, HDL and FBG, were measured biochemically within 3 h after the peripheral blood draw.

**Fig. 2:**
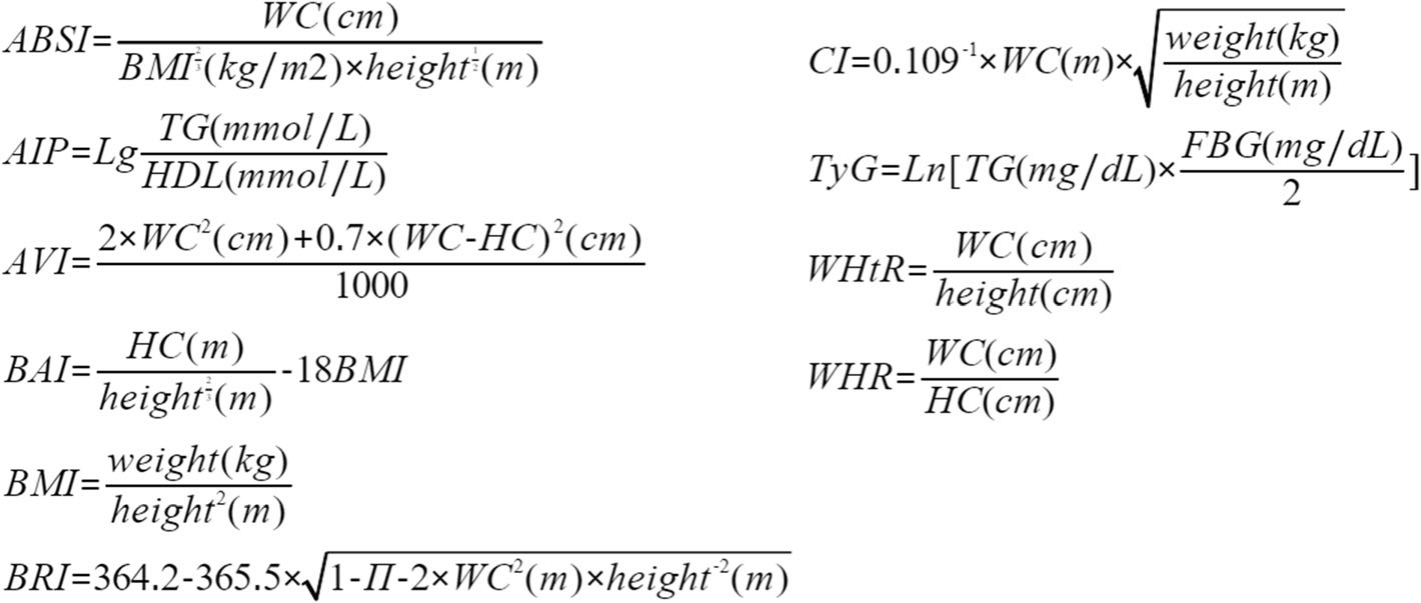
Equations of obesity-related indices

The obesity-related indices, including ABSI, AIP, AVI, BAI, BMI, BRI, CI, TyG, WHR, and WHtR, were calculated using the equations shown in Fig. 2 [20, 21, 25, 29].

Diagnoses of NAFLD were based on the “Chinese Guideline on Diagnosis and Treatment of NAFLD (2006)” by experienced radiologists with expertise in liver imaging. The NAFLD diagnosis met two of the three following items: diffuse hyperechoic liver relative to kidney, ultrasound beam attenuation, and weakening visualization of intrahepatic structures. In addition, patients with no history of drinking or an alcohol intake less than 40 g in males or 20 g in females per day over 5 years were included in this study [30].

### Statistical analysis

Categorical variables are expressed as numbers (percentages). Continuous variables are expressed as medians and two specific percentiles (P_25_ and P_75_) for nonnormally distributed data. All participants were divided into NAFLD and non-NAFLD groups. The baseline variables (sex, age, anthropometric and metabolic indices) were compared using the chi-square test and rank tests.

The values for SBP, DBP, FBG, TC, TG, LDL, WC, ABSI, AIP, AVI, BAI, BMI, BRI, CI, TyG, WHR and WHtR were divided into 4 quartiles according to their own changes. The first quartile was used as a reference. Logistic analyses were performed to determine the associations between these anthropometric and metabolic indices and NAFLD with odds ratios (ORs) and 95% confidence intervals (CIs).

Receiver operating characteristic (ROC) curves and areas under the curve (AUCs) were generated to compare the predictive ability of the various indices for identifying NAFLD.

All statistical analyses were performed with the Statistical Package for the Sciences software (SPSS, version 23.0). A value of *P* < 0.05 in the two-tailed test was considered significant.

## Results

### Characteristics of the study population

A total of 1748 subjects were included in this study, including 526 (30.09%) patients and 1222 (69.91%) control subjects. The mean ages of the patients and control subjects were 48.55 ± 14.21 and 46.18 ± 14.74 years, respectively (*P* < 0.01). Among these subjects, 464 (26.54%) males and 62 (3.55%) females had NAFLD. Table 1 compares the demographic characteristics and anthropometric and metabolic indices of the individuals in both groups. The percentage of NAFLD in males was higher than that in females, and the age of female subjects with NAFLD was significantly older than that of female subjects without NAFLD. Subjects with NAFLD had significantly higher SBP, DBP, FBG TC, TG, LDL, WC, ABSI, AIP, AVI, BAI, BMI, BRI, CI, TyG, WHR and WHtR than those without NAFLD in both the male and female groups (all *P* < 0.01).

**Table 1:** Demographic, anthropometric, and metabolic characteristics of the study participants

Characteristics	All (*n* = 1748)		Male (*n* = 1153)			Female (*n* = 595)			
	FL (+)	FL (−)	*P*-Value	FL (+)	FL (−)	*P*-Value	FL (+)	FL (−)	*P*-Value
**n (%)**	526 (30.09%)	1222 (69.91%)	< 0.01	464 (26.54%)	689 (39.42%)	< 0.01	62 (3.55%)	533 (30.49%)	< 0.01
**Age (years)**	46 (38, 57)	43 (34, 55)	< 0.01	45 (38, 56)	46 (36, 58)	0.368	55 (42, 65)	40 (33, 51)	< 0.01
**SBP (mmHg)**	130 (119, 144)	120 (108, 133)	< 0.01	130 (119, 143)	124 (114, 137)	< 0.01	132 (118, 151)	113 (104, 127)	< 0.01
**DBP (mmHg)**	80 (72, 87)	73 (65, 81)	< 0.01	80 (72, 88)	76 (68, 83)	< 0.01	77 (71, 84)	69 (62, 77)	< 0.01
**FBG (mmol/l)**	5.47 (5.07, 6.33)	5.10 (4.81, 5.46)	< 0.01	5.47 (5.07, 6.36)	5.17 (4.86, 5.58)	< 0.01	5.49 (5.15, 6.18)	5.02 (4.76, 5.33)	< 0.01
**TC (mmol/l)**	5.17 (4.54, 5.82)	4.89 (4.30, 5.54)	< 0.01	5.13 (4.53, 5.79)	4.90 (4.34, 5.62)	< 0.01	5.41 (4.61, 6.14)	4.89 (4.25, 5.47)	< 0.01
**TG (mmol/l)**	2.03 (1.41, 2.79)	1.11 (0.82, 1.57)	< 0.01	2.08 (1.46, 2.82)	1.21 (0.92, 1.74)	< 0.01	1.68 (1.35, 2.54)	0.96 (0.73, 1.33)	< 0.01
**LDL (mmol/l)**	3.26 (2.87, 3.75)	3.00 (2.54, 3.46)	< 0.01	3.24 (2.86, 3.67)	3.05 (2.63, 3.55)	< 0.01	3.43 (2.88, 3.92)	2.94 (2.47, 3.36)	< 0.01
**WC (cm)**	92.9 (88.0, 99.1)	82.0 (76.5, 87.8)	< 0.01	94.0 (88.6, 99.7)	84.2 (78.8, 90.1)	< 0.01	88.9 (85.6, 93.4)	78.8 (74.1, 84.0)	< 0.01
**ABSI**	0.081 (0.079, 0.083)	0.080 (0.078, 0.082)	< 0.01	0.081 (0.079, 0.083)	0.080 (0.077, 0.082)	< 0.01	0.081 (0.079, 0.083)	0.081 (0.079, 0.082)	0.334
**AIP**	0.59 (0.13, 0.98)	−0.18 (−0.56, 0.22)	< 0.01	0.63 (0.16, 0.99)	−0.03 (−0.037, 0.039)	< 0.01	0.33 (− 0.01, 0.72)	− 0.42 (− 0.76, 0)	< 0.01
**AVI**	17.3 (15.5, 19.7)	13.5 (11.8, 15.5)	< 0.01	17.7 (15.8, 19.9)	14.3 (12.5, 16.3)	< 0.01	15.8 (14.7, 17.4)	12.5 (11.1, 14.2)	< 0.01
**BAI**	27.3 (25.5, 20.3)	25.5 (23.8, 27.3)	< 0.01	27.0 (25.3, 28.9)	24.6 (22.9, 26.3)	< 0.01	29.8 (27.9, 31.9)	26.7 (24.8, 28.5)	< 0.01
**BMI**	26.5 (24.6, 28.5)	22.7 (20.5, 24.5)	< 0.01	26.7 (24.7, 28.6)	23.4 (21.5, 24.9)	< 0.01	25.5 (23.9, 26.9)	21.6 (19.9, 23.5)	< 0.01
**BRI**	4.36 (3.77, 5.00)	3.22 (2.72, 3.84)	< 0.01	4.32 (3.73, 5.00)	3.24 (2.75, 3.84)	< 0.01	4.52 (4.11, 5.01)	21.6 (19.9, 23.5)	< 0.01
**CI**	1.28 (1.25, 1.31)	1.23 (1.20, 1.27)	< 0.01	1.27 (1.24, 1.32)	1.23 (1.20, 1.27)	< 0.01	1.28 (1.25, 1.31)	1.23 (1.21, 1.26)	< 0.01
**TyG**	4.90 (4.71, 5.10)	4.56 (4.40, 4.75)	< 0.01	4.92 (4.72, 5.11)	4.62 (4.47, 4.81)	< 0.01	4.83 (4.66, 5.09)	4.47 (4.32, 4.67)	< 0.01
**WHR**	0.93 (0.90, 0.96)	0.88 (0.85, 0.91)	< 0.01	0.93 (0.90, 0.96)	0.88 (0.85, 0.92)	< 0.01	0.93 (0.90, 0.95)	0.87 (0.84, 0.90)	< 0.01
**WHtR**	0.55 (0.52, 0.58)	0.49 (0.46, 0.53)	< 0.01	0.55 (0.52, 0.58)	0.49 (0.47, 0.53)	< 0.01	0.56 (0.54, 0.58)	0.49 (0.46, 0.53)	< 0.01

### ORs for NAFLD risk across quartiles of each index

Table 2 demonstrates that the analyzed parameters were significantly associated with NAFLD (*P* < 0.01). The ORs for NAFLD still increased across the quartiles of each index in males and females after adjusting for sex and age. Among all subjects, the BMI showed the highest risk of NAFLD among all the indices, followed by WC and AVI.

**Table 2:** ORs for NAFLD stratified by quartiles of each index

Variables	Constant	Q1	Q2		Q3		Q4		*P*
	B	OR (CI)	B	OR (CI)	B	OR (CI)	B	OR (CI)	
**Crude model**									
**SBP (mmHg)**	−1.91	1	0.99	2.69 (1.91, 3.79) **	1.33	3.79 (2.71, 5.29) **	1.68	5.38 (3.86, 7.50) **	< 0.01
**DBP (mmHg)**	−1.75	1	0.79	2.21 (1.59, 3.08) **	1.06	2.90 (2.09, 4.01) **	1.60	4.97 (3.61, 6.85) **	< 0.01
**FBG (mmol/l)**	−1.55	1	0.29	1.34 (0.96, 1.86)	0.66	1.92 (1.40, 2.66) **	1.64	5.12 (3.77, 6.98) **	< 0.01
**TC (mmol/l)**	−1.23	1	0.28	1.32 (0.97, 1.79)	0.54	1.71 (1.27, 2.31) **	0.70	2.01 (1.50, 2.71) **	< 0.01
**TG (mmol/l)**	−2.66	1	1.15	3.15 (2.01, 4.94) **	2.04	7.68 (5.02, 11.74) **	3.13	22.94 (15.02, 35.02) **	< 0.01
**LDL (mmol/l)**	−1.49	1	0.61	1.84 (1.34, 2.52) **	0.80	2.22 (1.62, 3.03) **	1.07	2.92 (2.15, 3.98) **	< 0.01
**WC (cm)**	−3.67	1	1.74	5.71 (2.95, 11.08) **	3.26	25.92 (13.83, 18.58) **	4.31	74.21 (39.52, 139.35) **	< 0.01
**ABSI**	−1.24	1	0.36	1.43 (1.06, 1.94) *	0.46	1.58 (1.17, 2.14) **	0.71	2.03 (1.51, 2.74) **	< 0.01
**AIP**	−3.12	1	1.61	5.00 (2.97, 8.41) **	2.56	12.87 (7.81, 21.21) **	3.64	38.23 (23.21, 62.96) **	< 0.01
**AVI**	−3.66	1	1.76	5.82 (3.00, 11.27) **	3.25	25.68 (13.70, 48.14) **	4.28	72.53 (38.63, 136.19) **	< 0.01
**BAI**	−2.02	1	0.97	2.64 (1.84, 3.79) **	1.40	4.04 (2.84, 5.74) **	1.95	7.00 (4.95, 9.91) **	< 0.01
**BMI**	−4.12	1	2.55	12.79 (5.82, 28.09) **	3.45	31.50 (14.55, 68.20) **	4.89	132.37 (61.07, 286.93) **	< 0.01
**BRI**	−3.34	1	1.78	5.92 (3.35, 10.49) **	2.75	15.62 (9.00, 27.10) **	3.91	49.68 (28.64, 86.17) **	< 0.01
**CI**	−2.22	1	0.69	1.98 (1.30, 3.03) **	1.67	5.30 (3.52, 7.98) **	2.40	11.02 (7.34, 16.53) **	< 0.01
**TyG**	−2.85	1	1.32	3.72 (2.34, 6.00) **	2.30	9.97 (6.37, 15.62) **	3.38	29.45 (18.81, 46.14) **	< 0.01
**WHR**	−2.54	1	1.20	3.31 (2.23, 4.95) **	2.37	10.66 (7.17, 15.86) **	2.81	16.55 (11.29, 24.24) **	< 0.01
**WHtR**	−3.82	1	1.81	6.12 (3.54, 10.56) **	2.90	18.17 (10.64, 31.02) **	3.95	51.95 (30.14, 89.54) **	< 0.01
**After adjusting gender and age**									
**SBP (mmHg)**	−2.55	1	0.72	2.06 (1.44, 2.94) **	1.03	2.80 (1.97, 3.97) **	1.42	4.14 (2.85, 6.00) **	< 0.01
**DBP (mmHg)**	−2.70	1	0.61	1.85 (1.31, 2.60) **	0.77	2.16 (1.53, 3.04) **	3.36	2.39 (2.39, 4.72) **	< 0.01
**FBG (mmol/l)**	−2.26	1	0.30	1.35 (0.95, 1.91)	0.63	1.88 (1.34, 2.64) **	1.62	5.04 (3.56, 7.13) **	< 0.01
**TC (mmol/l)**	−2.80	1	0.22	1.24 (0.90, 1.71)	0.58	1.79 (1.31, 2.46) **	0.65	1.92 (1.40, 2.62) **	< 0.01
**TG (mmol/l)**	−3.60	1	0.92	2.52 (1.59, 3.98) **	1.77	5.89 (3.82, 9.09) **	2.79	16.32 (10.59, 25.15) **	< 0.01
**LDL (mmol/l)**	−3.01	1	0.52	1.68 (1.21, 2.35) **	0.75	2.11 (1.52, 2.92) **	0.93	2.53 (1.84, 3.50) **	< 0.01
**WC (cm)**	−5.15	1	1.62	5.01 (2.57, 9.78) **	3.05	21.17 (11.20, 40.01) **	4.06	58.17 (30.57, 110.66) **	< 0.01
**ABSI**	−3.27	1	0.64	1.89 (1.37, 2.62) **	0.76	2.13 (1.54, 2.95) **	1.01	2.74 (1.99, 3.76) **	< 0.01
**AIP**	−3.90	1	1.37	3.92 (2.31, 6.64) **	2.24	9.41 (5.66, 15.63) **	3.24	25.45 (15.32, 42.29) **	< 0.01
**AVI**	−5.16	1	1.63	5.09 (2.61, 9.91) **	3.04	20.93 (11.07, 39.58) **	4.04	57.04 (29.97, 108.56) **	< 0.01
**BAI**	−4.06	1	1.18	3.26 (2.25, 4.74) **	1.86	6.40 (4.41, 9.28) **	2.77	15.97 (10.80, 23.62) **	< 0.01
**BMI**	−5.28	1	2.33	10.23 (4.64, 22.58) **	3.12	22.74 (10.45, 49.49) **	4.56	95.98 (44.06, 209.10) **	< 0.01
**BRI**	−4.93	1	1.70	5.49 (3.08, 9.80) **	2.64	14.00 (8.00, 24.47) **	3.92	50.41 (28.71, 88.53) **	< 0.01
**CI**	−4.21	1	0.90	2.47 (1.59, 3.83) **	1.83	6.25 (4.08, 9.57) **	2.50	12.21 (7.99, 18.67) **	< 0.01
**TyG**	−3.39	1	1.09	2.97 (1.84, 4.80) **	2.06	7.83 (4.95, 12.39) **	3.05	21.07 (13.27, 33.46) **	< 0.01
**WHR**	−4.03	1	1.14	3.13 (2.08, 4.71) **	2.27	9.66 (6.41, 14.55) **	2.64	14.07 (9.50, 20.84)	< 0.01
**WHtR**	−4.84	1	1.73	5.88 (3.26, 9.85) **	2.83	16.88 (9.80, 29.07) **	3.95	51.69 (29.60, 60.25)	< 0.01

* *P*-value < 0.05. ** *P*-value < 0.01

### ROC curves and AUC for indices in identifying NAFLD

Table 3 shows that BMI had the greatest AUC of 0.841. WC and AVI showed the same ability for predicting NAFLD and had the second highest AUC (0.836) among all the indices. The Youden index values of these indices were over 0.5.

**Table 3:** AUC, Youden index, sensitivity, specificity and cut-off point of clinical parameters and obesity-related indices for predicting NAFLD

	AUC (95% CI)	Sensitivity (%)	Specificity (%)	Youden Index	Cut-off point
**SBP**	0.660 (0.634, 0.687) **	68.3	56.5	0.247	122.5
**DBP**	0.663 (0.636, 0.690) **	81.4	57.0	0.243	70.5
**FBG**	0.682 (0.654, 0.710) **	59.7	67.7	0.274	5.32
**TC**	0.581 (0.552, 0.610) **	65.6	47.7	0.132	4.82
**TG**	0.785 (0.762, 0.808) **	73.0	71.5	0.445	1.48
**LDL**	0.610 (0.582, 0.638) **	76.4	42.0	0.184	2.86
**WC**	0.836 (0.816, 0.855) **	87.5	66.1	0.536	85.3
**ABSI**	0.578 (0.549, 0.607) **	63.7	48.9	0.125	0.0799
**AIP**	0.804 (0.783, 0.825) **	80.8	65.4	0.462	0.045
**AVI**	0.836 (0.817, 0.855) **	87.5	66.0	0.535	14.6
**BAI**	0.691 (0.665, 0.717) **	64.8	64.0	0.288	26.4
**BMI**	0.841 (0.822, 0.860) **	71.3	80.1	0.521	24.9
**BRI**	0.817 (0.796, 0.837) **	78.3	70.1	0.485	3.67
**CI**	0.740 (0.715, 0.765) **	68.6	70.1	0.388	1.26
**TyG**	0.807 (0.785, 0.828) **	72.1	74.5	0.466	4.75
**WHR**	0.776 (0.754, 0.799) **	73.6	70.8	0.444	0.905
**WHtR**	0.815 (0.794, 0.836) **	78.7	69.1	0.478	0.515

* *P*-value < 0.05. ** *P*-value < 0.01

**Fig. 3:**
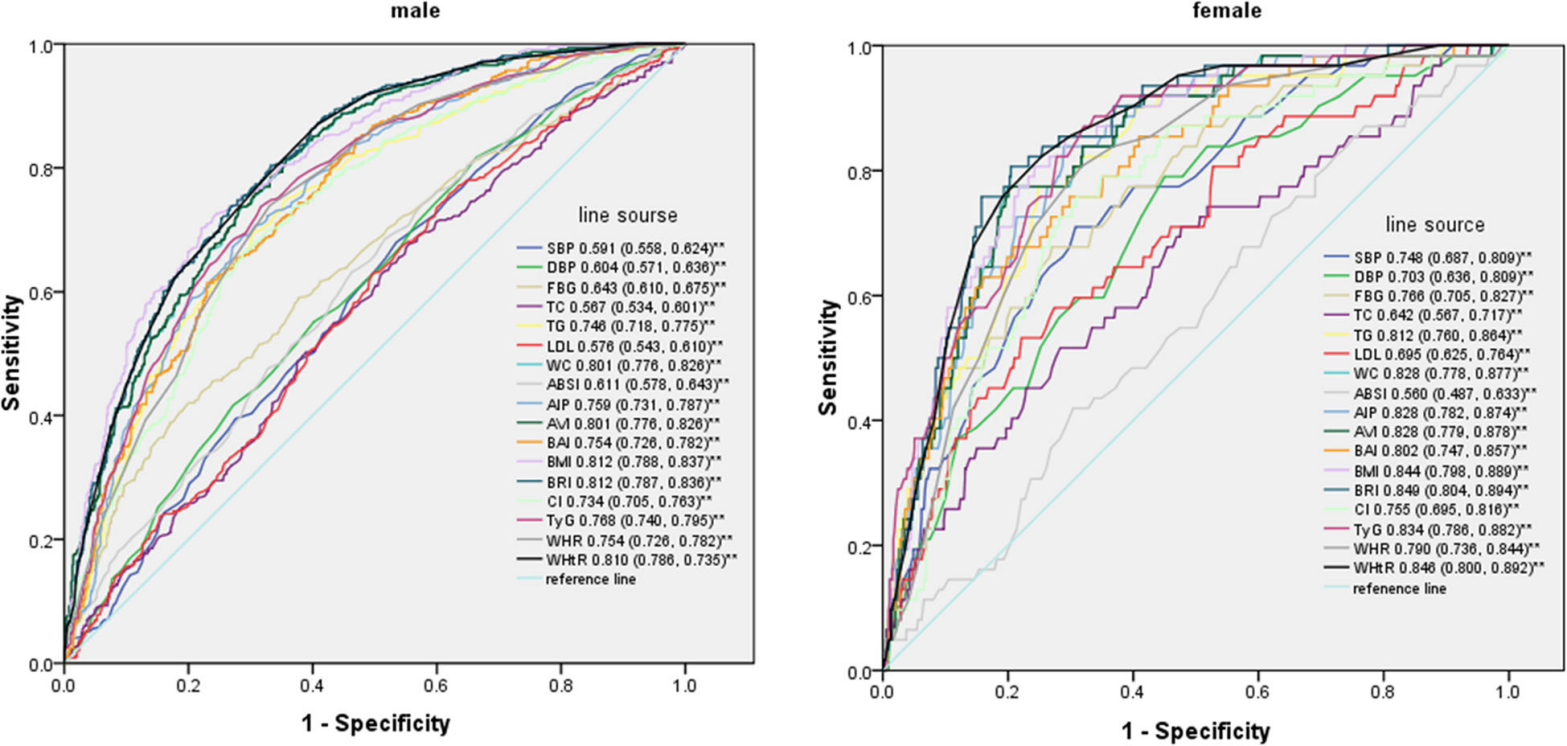
Comparison of the predictive value of NAFLD-related parameters for diagnosis of NAFLD among males and females

As shown in Fig. 3, BMI and BRI had the same diagnostic ability for NAFLD in males (AUC = 0.812), and WHtR had the next best diagnostic ability (AUC = 0.810). In females, BRI also had the greatest predictive ability (AUC = 0.849), and WHtR (AUC = 0.846) had the second greatest predictive ability, followed by BMI (AUC = 0.844).

**Fig. 4:**
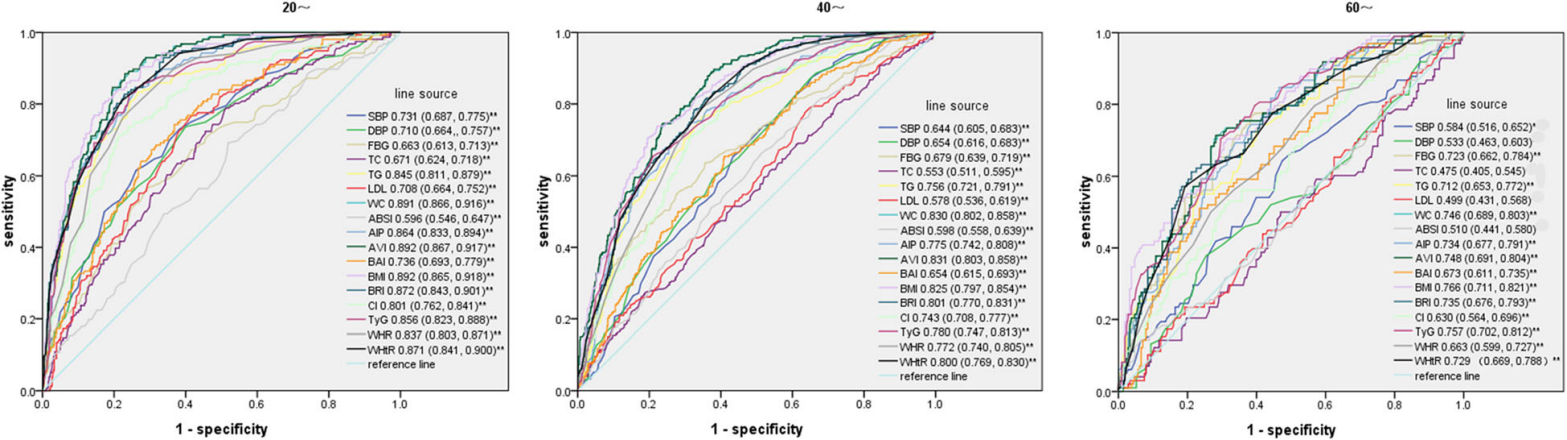
Comparison of the predictive value of NAFLD-related parameters for diagnosis of FL among ~ 20, ~ 40 and ~ 60 years old

Figure 4 presents the ROC curves and AUCs of the indices for NAFLD in patients who were ~ 20, ~ 40 and ~ 60 years old. For all three age groups, BMI and WC were both in the top three indices for predictive ability. AVI had the greatest AUC in the ~ 20 years age group (AUC = 0.892) and the ~ 40 years age group (AUC = 0.831), while TyG had a higher AUC than AVI in the ~ 60 years age group (AUC = 0.757). In addition, DBP, TC, LDL and ABSI in subjects aged ~ 60 had no significant predictive ability (*P* > 0.05).

## Discussion

This cross-sectional study comprehensively evaluated the predictive ability and cutoff value of obesity-related anthropometric and metabolic indices to identify NAFLD and found that BMI had the greatest AUC among all participants. Additionally, sex- and age-specific indices for predicting NAFLD existed.

Age and sex might be critical factors affecting the prevalence of NAFLD [3, 4, 11]. This study found that men had a similar prevalence of NAFLD regardless of age, whereas it increased steadily with age in women. This is also consistent with a previous finding that aging is a risk factor for NAFLD in Japanese women, independent of weight gain or the influence of metabolic syndrome [31]. The increased prevalence of NAFLD with age for females might be associated with alterations in sex hormones postmenopause. Visceral adiposity may be caused by the loss of estrogen after menopause, which may lead to extensive changes in the metabolic system. Generally, NAFLD is primarily considered a male disease; however, the alteration in sex hormone levels, specifically reduced estrogens and increased androgens during and after menopause, might play an important role in the emergence of NAFLD in female subjects [32, 33]. Investigators should also pay attention to NAFLD with increased age in Chinese females.

Epidemiological studies propose a causative link between obesity and progressive liver disease in individuals, and this association was observed at both the initial stages and severe stages of the disease [18, 34]. Pathophysiological and clinical studies have shown that an imbalance between lipid uptake and lipid utilization may eventually cause oxidative stress and hepatocyte injury [35]. Some studies have suggested that visceral adiposity is the main adipose depot responsible for NAFLD, and visceral adiposity was associated with NAFLD in a dose-dependent manner in a cohort study [36]. WC, WHR, WHtR and BMI have been used in many clinical trials as indicators of the severity of fatty liver disease [21, 24]. The calculation of the ABSI considers the adjustment for height and WC compared with BMI [37]. AVI is used to assess general volume, and it has been highly associated with the dysfunction of glucose metabolism [38]. Increased AIP might be concordantly associated with the incidence of NAFLD [19, 20]. BAI is a better and more easily applicable measure for the determination of body fat than BMI, WHR, WHtR and WC in Turkish adults [39]. BRI was used to predict body fat and the percentage of visceral adipose tissue by Thomas et al. [40]. TyG is often used to explore the relationship between insulin resistance and excessive visceral fat accumulation [41, 42]. Additionally, accumulating evidence strongly suggests that advanced blood lipids, blood pressure and blood sugar could also lead to more severe histological changes and poorer clinical outcomes [17, 24, 43]. Furthermore, insulin resistance can promote the progression of NAFLD to a more severe state of liver endangerment, such as nonalcoholic steatohepatitis.

This study also revealed that BMI and BRI had a relatively high association with NAFLD and high diagnostic ability (0.812 in men and 0.849 in women) for NAFLD after considering the influence of sex. These results are also supported by research by Nima Motamed et al., which reported high AUCs for BRI and WHtR (0.85 in men and 0.86 in women) [44]. The subtle differences between the two studies may be due to the differences between Chinese and Iranian populations. The findings that AVI had the greatest AUC in the ~ 20 and ~ 40 age groups are concurrent with the study by Filippo Procino et al. They reported that AVI had a low false negative rate and high ability to identify NAFLD, and it should be mentioned that their research may be more helpful in NAFLD prediction for people in the 20–59 age range [45]. Diabetes is one of the strongest risk factors for NAFLD, and the increasing prevalence of diabetes with age, especially in female subjects [15, 46]. These findings may explain the result that AVI had the greatest AUC in the ~ 20 and ~ 40 years old age groups, while TyG had a higher AUC than AVI in the ~ 60 years old age group.

### Strengths and limitations of the study

One of the biggest strengths of this study is that almost all obesity-related anthropometric and metabolic indices were included in this study to be comprehensively evaluated by sex and age. Although the association between these obesity-related indices and NAFLD has been analyzed separately in many articles, few articles have combined them for evaluation, especially in a Chinese population.

However, there are still some limitations. First, this is a cross-sectional study. Second, the data of other confounders, such as smoking and drinking status and exercise, were not included in the analysis because this information was not available. Third, ultrasonography diagnosis is a fast, reliable, reproducible and invasive method compared with liver biopsy, but it is unable to adequately determine the levels of steatosis and fibrosis.

## Conclusion

This study found sex- and age-specific indices for predicting NAFLD in Chinese subjects. For the population as a whole, BMI might be the best predictor for NAFLD, followed by WC and AVI. When stratified by sex, BRI and BMI might both be the best predictors for NAFLD in males, and BRI was suitable for predicting NAFLD in females. Considering age, AVI had the greatest AUC for those aged 20–60 years, while TyG had the higher predictive ability in those ~ 60 years old. Compared with indices for all age groups, sex- and age-specific indices can provide more accurate assistance for clinical diagnosis and treatment. In addition, clues of disease cause can be found by comparing sex- and age-specific indices.

## Supplementary Information

**Additional file 1.**

**Additional file 2.**

## Data Availability

The datasets generated and analysed during the current study are not publicly available due personal privacy but are available from the corresponding author on reasonable request.

## Abbreviations

NAFLD: Non-alcoholic Fatty Liver Disease
SBP: Systolic Blood Pressure
DBP: Diastolic Blood Pressure
FBG: Fasting Blood Glucose
TC: Total Cholesterol
TG: Triglyceride
LDL: Low Density Lipoprotein
WC: Waist Circumference
ABSI: A Body Shape Index
AIP: Atherogenic Index of Plasma
AVI: Abdominal Volume Index
BAI: Body Adiposity Index
BMI: Body Mass Index
BRI: Body Roundness Index
CI: Conicity Index
TyG: Triglyceride Glucose
WHR: Waist–hip Ratio
WHtR: Waist-to-Height Ratio
OR: Odds Ratio
ROC: Receiver Operating Characteristic
AUC: Areas Under Curve

## References

[1] Liu K, McCaughan GW (2018). Epidemiology and etiologic associations of non-alcoholic fatty liver disease and associated HCC. Adv Exp Med Biol.

[2] Leoni S, Tovoli F, Napoli L, Serio I, Ferri S, Bolondi L (2018). Current guidelines for the management of non-alcoholic fatty liver disease: a systematic review with comparative analysis. World J Gastroenterol.

[3] Araujo AR, Rosso N, Bedogni G, Tiribelli C, Bellentani S (2018). Global epidemiology of non-alcoholic fatty liver disease/non-alcoholic steatohepatitis: what we need in the future. Liver Int.

[4] Andronescu CI, Purcarea MR, Babes PA (2018). Nonalcoholic fatty liver disease: epidemiology, pathogenesis and therapeutic implications. J Med Life.

[5] Perumpail BJ, Khan MA, Yoo ER, Cholankeril G, Kim D, Ahmed A (2017). Clinical epidemiology and disease burden of nonalcoholic fatty liver disease. World J Gastroenterol.

[6] Sanyal AJ (2019). Past, present and future perspectives in nonalcoholic fatty liver disease. Nat Rev Gastroenterol Hepatol.

[7] Marchisello S, Di Pino A, Scicali R, Urbano F, Piro S, Purrello F, et al. Pathophysiological, molecular and therapeutic issues of nonalcoholic fatty liver disease: an overview. Int J Mol Sci. 2019;20(8). 10.3390/ijms20081948. 10.3390/ijms20081948 PMC6514656 31010049

[8] Farzanegi P, Dana A, Ebrahimpoor Z, Asadi M, Azarbayjani MA (2019). Mechanisms of beneficial effects of exercise training on non-alcoholic fatty liver disease (NAFLD): roles of oxidative stress and inflammation. Eur J Sport Sci.

[9] Hernandez-Rodas MC, Valenzuela R, Videla LA (2015). Relevant aspects of nutritional and dietary interventions in non-alcoholic fatty liver disease. Int J Mol Sci.

[10] Valenzuela R, Videla LA (2011). The importance of the long-chain polyunsaturated fatty acid n-6/n-3 ratio in development of non-alcoholic fatty liver associated with obesity. Food Funct.

[11] Wijarnpreecha K, Panjawatanan P, Aby E, Ahmed A, Kim D (2019). Nonalcoholic fatty liver disease in the over-60s: impact of sarcopenia and obesity. Maturitas.

[12] Agbim U, Carr RM, Pickett-Blakely O, Dagogo-Jack S (2019). Ethnic disparities in adiposity: focus on non-alcoholic fatty liver disease, visceral, and generalized obesity. Curr Obes Rep.

[13] Mantovani A, Byrne CD, Bonora E, Targher G (2018). Nonalcoholic fatty liver disease and risk of incident type 2 diabetes: a Meta-analysis. Diabetes Care.

[14] Polyzos SA, Kountouras J, Mantzoros CS (2017). Adipose tissue, obesity and non-alcoholic fatty liver disease. Minerva Endocrinol.

[15] Chao HW, Chao SW, Lin H, Ku HC, Cheng CF. Homeostasis of glucose and lipid in non-alcoholic fatty liver disease. Int J Mol Sci. 2019;20(2). 10.3390/ijms20020298. 10.3390/ijms20020298 PMC6359196 30642126

[16] Targher G, Li Y, Wang J, Tang Y, Han X, Liu B, Hu H, Li X, Yang K, Yuan J (2017). Bidirectional association between nonalcoholic fatty liver disease and type 2 diabetes in Chinese population: evidence from the Dongfeng-Tongji cohort study. PLoS One.

[17] Arrese M, Barrera F, Triantafilo N, Arab JP (2019). Concurrent nonalcoholic fatty liver disease and type 2 diabetes: diagnostic and therapeutic considerations. Expert Rev Gastroenterol Hepatol.

[18] Li L, Liu DW, Yan HY, Wang ZY, Zhao SH, Wang B (2016). Obesity is an independent risk factor for non-alcoholic fatty liver disease: evidence from a meta-analysis of 21 cohort studies. Obes Rev.

[19] Xie F, Zhou H, Wang Y (2019). Atherogenic index of plasma is a novel and strong predictor associated with fatty liver: a cross-sectional study in the Chinese Han population. Lipids Health Dis.

[20] Wang Q, Zheng D, Liu J, Fang L, Li Q (2018). Atherogenic index of plasma is a novel predictor of non-alcoholic fatty liver disease in obese participants: a cross-sectional study. Lipids Health Dis.

[21] VanWagner LB, Khan SS, Ning H, Siddique J, Lewis CE, Carr JJ, Vos MB, Speliotes E, Terrault NA, Rinella ME (2018). Body mass index trajectories in young adulthood predict non-alcoholic fatty liver disease in middle age: the CARDIA cohort study. Liver Int.

[22] Chen Z, Qin H, Qiu S, Chen G, Chen Y (2019). Correlation of triglyceride to high-density lipoprotein cholesterol ratio with nonalcoholic fatty liver disease among the non-obese Chinese population with normal blood lipid levels: a retrospective cohort research. Lipids Health Dis.

[23] Rachakonda V, Wills R, DeLany JP, Kershaw EE, Behari J (2017). Differential impact of weight loss on nonalcoholic fatty liver resolution in a north American cohort with obesity. Obesity (Silver Spring).

[24] Ren XY, Shi D, Ding J, Cheng ZY, Li HY, Li JS, Pu HQ, Yang AM, He CL, Zhang JP, Ma YB, Zhang YW, Zheng TZ, Bai YN, Cheng N (2019). Total cholesterol to high-density lipoprotein cholesterol ratio is a significant predictor of nonalcoholic fatty liver: Jinchang cohort study. Lipids Health Dis.

[25] Li Y, Zheng R, Li J, Feng S, Wang L, Huang Z (2020). Association between triglyceride glucose-body mass index and non-alcoholic fatty liver disease in the non-obese Chinese population with normal blood lipid levels: a secondary analysis based on a prospective cohort study. Lipids Health Dis.

[26] Guo W, Lu J, Qin P, Li X, Zhu W, Wu J, Xu N, Zhang Q (2020). The triglyceride-glucose index is associated with the severity of hepatic steatosis and the presence of liver fibrosis in non-alcoholic fatty liver disease: a cross-sectional study in Chinese adults. Lipids Health Dis.

[27] Wang Y, Zeng Y, Lin C, Chen Z (2016). Hypertension and non-alcoholic fatty liver disease proven by transient elastography. Hepatol Res.

[28] Pan XF, Wang L, Pan A (2021). Epidemiology and determinants of obesity in China. Lancet Diabetes Endocrinol.

[29] Guerrero-Romero F, Rodríguez-Morán M. Abdominal volume index. an anthropometry-based index for estimation of obesity is strongly related to impaired glucose tolerance and type 2 diabetes mellitus. Arch Med Res. 2003;34(5):428–32. 10.1016/S0188-4409(03)00073-0 14602511

[30] Fan JG (2007). An introduction of strategies for the management of nonalcoholic fatty liver disease (NAFLD) recommended by Asia Pacific working party on NAFLD. Zhonghua Gan Zang Bing Za Zhi.

[31] Hamaguchi M, Kojima T, Ohbora A, Takeda N, Kato T (2012). Aging is a risk factor of nonalcoholic fatty liver disease in premenopausal women. World J Gastroenterol.

[32] Hörist-Kollmann S, Strametz-Juranek J (2018). Female dietary patterns and the pathogenesis of NAFLD. Gend Genome.

[33] Cai M-J, Kong X-N, Zhao X-Y (2017). Influences of gender and age on the prevalence and complications of nonalcoholic fatty liver disease. Acta Academiae Medicinae Sinicae.

[34] Mahli A, Hellerbrand C (2016). Alcohol and obesity: a dangerous Association for Fatty Liver Disease. Dig Dis.

[35] Cholankeril G, Wong RJ, Hu M, Perumpail RB, Yoo ER, Puri P, Younossi ZM, Harrison SA, Ahmed A (2017). Liver transplantation for nonalcoholic steatohepatitis in the US: temporal trends and outcomes. Dig Dis Sci.

[36] Kim D, Chung GE, Kwak MS, Seo HB, Kang JH, Kim W, Kim YJ, Yoon JH, Lee HS, Kim CY (2016). Body fat distribution and risk of incident and regressed nonalcoholic fatty liver disease. Clin Gastroenterol Hepatol.

[37] Christakoudi S, Tsilidis KK, Muller DC, Freisling H, Weiderpass E, Overvad K, Soderberg S, Haggstrom C, Pischon T, Dahm CC (2020). A body shape index (ABSI) achieves better mortality risk stratification than alternative indices of abdominal obesity: results from a large European cohort. Sci Rep.

[38] Quaye L, Owiredu W, Amidu N, Dapare PPM, Adams Y (2019). Comparative abilities of body mass index, waist circumference, abdominal volume index, body adiposity index, and Conicity index as predictive screening tools for metabolic syndrome among apparently healthy Ghanaian adults. J Obes.

[39] Yeşil E, Köse B, Özdemir M (2020). Is body adiposity index a better and easily applicable measure for determination of body fat?. J Am Coll Nutr.

[40] Thomas DM, Bredlau C, Bosy-Westphal A, Mueller M, Shen W, Gallagher D, Maeda Y, McDougall A, Peterson CM, Ravussin E, Heymsfield SB (2013). Relationships between body roundness with body fat and visceral adipose tissue emerging from a new geometrical model. Obesity (Silver Spring).

[41] Umano GR, Shabanova V, Pierpont B, Mata M, Nouws J, Trico D, Galderisi A, Santoro N, Caprio S (2019). A low visceral fat proportion, independent of total body fat mass, protects obese adolescent girls against fatty liver and glucose dysregulation: a longitudinal study. Int J Obes.

[42] Nirengi S, Fujibayashi M, Furuno S, Uchibe A, Kawase Y, Sukino S, Kawaguchi Y, Minato S, Kotani K, Sakane N (2018). Nonalcoholic Fatty Liver Disease in University Rugby Football Players. Front Endocrinol (Lausanne).

[43] Oikonomou D, Georgiopoulos G, Katsi V, Kourek C, Tsioufis C, Alexopoulou A, Koutli E, Tousoulis D (2018). Non-alcoholic fatty liver disease and hypertension: coprevalent or correlated?. Eur J Gastroenterol Hepatol.

[44] Motamed N, Rabiee B, Hemasi GR, Ajdarkosh H, Khonsari MR, Maadi M, Keyvani H, Zamani F (2016). Body roundness index and waist-to-height ratio are strongly associated with non-alcoholic fatty liver disease: a population-based study. Hepat Mon.

[45] Zheng RD, Chen ZR, Chen JN, Lu YH, Chen J (2012). Role of body mass index, Waist-to-Height and Waist-to-Hip Ratio in Prediction of Nonalcoholic Fatty Liver Disease. Gastroenterol Res Pract.

[46] Harsha Varma S, Tirupati S, Pradeep TVS, Sarathi V, Kumar D (2019). Insulin resistance and hyperandrogenemia independently predict nonalcoholic fatty liver disease in women with polycystic ovary syndrome. Diabetes Metab Syndr.

