# Supplementary material for: Comparison of obesity-related indices for identifying nonalcoholic fatty liver disease: a population-based cross-sectional study in China
Source: Lipids Health Dis. 2021 Oct 10;20:132. doi: 10.1186/s12944-021-01560-3 (PMC8502306; doi:10.1186/s12944-021-01560-3)
Supplement: Supplementary file 2 — Additional file 2. [file 12944_2021_1560_MOESM2_ESM.pdf]

NAFLD

WORD COUNT 4523
TIME SUBMITTED 14-AUG-2021 06:07PM
PAPER ID 75545119

Comparison of Obesity Related Indices for Identifying Non-alcoholic Fatty Liver Disease: A Population-based Cross-sectional Study in China

Yuyu Pei[1#], Quan Zhou[1#], Deli Cao[1], Yun Wang[1], Fangfei Xie*[1]

1. Physical Examination Center, the Affiliated Suzhou Hospital of Nanjing Medical University, Suzhou, Jiangsu, P. R. China

[#] Yuyu Pei and Quan Zhou contributed equally to the writing of this article.

*Corresponding Author: Fangfei Xie, Mrs.

Physical Examination Center

the Affiliated Suzhou Hospital of Nanjing Medical University

Suzhou, Jiangsu, P. R. China

Tel:86-13913186601

E-mail: fangfeixie@hotmail.com

Abstract

Background: The relationship between Non-alcoholic Fatty Liver Disease (NAFLD) and obesity related indices had been analyzed separately presently, evidence existed for comparing those indexes together is still lacking, especially in China. This study aimed to comprehensively evaluate the predictive performance of anthropometric and metabolic indices in identifying NAFLD in Chinese adults.

Methods: We recruited a total of 1748 participants aged over 18 years in southeast of China. Systolic Blood Pressure (SBP), Diastolic Blood Pressure (DBP), Fasting Blood Glucose (FBG), Total Cholesterol (TC), Triglyceride (TG), Low Density Lipoprotein (LDL), Waist Circumference (WC), A Body Shape Index (ABSI), Atherogenic Index of Plasma (AIP), Abdominal Volume Index (AVI), Body Adiposity Index (BAI), Body Mass Index (BMI), Body Roundness Index (BRI), Conicity Index (CI), Triglyceride Glucose (TyG), Waist Hip Ratio (WHR), Waist Height Ratio (WHtR) were selected. The association between these indices and NAFLD was analyzed via logistic analyses with Odds Ratio (ORs). Receiver Operating Characteristic (ROC) curves and Areas Under Curves (AUCs) were used to compare the predictive performance of these indices to identify NAFLD.

Results: BMI had the greatest AUC in total (AUC = 0.841) in ROC curves analysis. However, BRI and BMI both had the first diagnostic ability in males (AUC = 0.812) and BRI had the first diagnostic ability in females (AUC = 0.849). Furthermore, AVI had the greatest AUC in 20~ (AUC = 0.892) and 40~ years (AUC = 0.831), while TyG showed higher predicting ability than AVI in 60~ years (AUC = 0.766).

Conclusion: We found sex and age-specific indices for predicting NAFLD in Chinese subjects. Compared with indices for all-age groups, sex and age-specific indices can provide more accurate assistance for clinical diagnosis and treatment.

Keywords: Non-alcoholic Fatty Liver Disease, Obesity, Indices, Anthropometric, Metabolic, Predict, Diagnostic ability

Background

It is well-established that Non-alcoholic Fatty Liver Disease (NAFLD) had become a major public

health problem over the past few decades with the incidences around 30% and 25% in Western and Asia countries, respectively. NAFLD have also exhibited huge medical and economic burden for both developed and developing countries[1-5]. NAFLD is also characterized by complex pathogenesis and difficulty in diagnosis[6, 7]. Thus, it is of great necessity to further explore the pathogenesis or effective predictive indicators for the diagnosis of NAFLD, which is critical for the prevention and treatment of NAFLD.

Oxidative stress and inflammation can promote NAFLD to non-alcoholic steatohepatitis (NASH), or even hepatic cirrhosis in the progression of NAFLD[8]. Although the pathogenesis of NAFLD is still not fully understood, obesity has been demonstrated to play a major role in most of the pathogenic pathways involved in NAFLD. Dietary nutrients play an increasingly important role in the progression of NAFLD in recent years via affecting the lipid and carbohydrate metabolism. For example, obesogenic diet is associated with hepatic oxidative stress and inflammation, which might be owing to the activation in anabolic pathways, and can eventually lead to abdominal obesity[9]. In contrast, the intake of polyunsaturated fatty acids (n-3 Polyunsaturated Fatty Acids) can reduce nutritional hepatic steatosis in adults which favor fatty acids and TGs formation over fatty acids oxidation[10].

NAFLD is commonly associated with visceral adiposity, type II diabetes, dyslipidemia, and metabolic disorders[11-15]. The relationship between NAFLD and type II diabetes is complex and bidirectional and occurs in the context of a wider association between NAFLD and metabolic syndrome[16, 17]. In the last decades, the prevalence of NAFLD has an alarming increase, along with increasing rates of obesity[18]. Some studies found that the development of NAFLD may be influenced by regional distribution of lean and fat mass and suggested that abdominal fat is a risk factor for both fatty liver and fatty liver fibrosis[7, 14]. Therefore, obesity associated factors might be utilized for predicting NAFLD.

As expected, several anthropometric or metabolic indices such as atherogenic index[19, 20], Body Mass Index (BMI)[21], Triglyceride (TG) / High-Density Lipoprotein cholesterol[22], visceral adipose tissue[23], Total cholesterol (TC) / High-Density Lipoprotein cholesterol[24] and Triglyceride Glucose (TyG)[25, 26], as well as blood pressure[27] have been reported to be associated with NAFLD in both cross-sectional and cohort studies. However, most existing studies

mainly focused on only one or two indices, which might have limitations for predicting NAFLD considering the high complexity of the pathogenesis of NAFLD. Meanwhile, it remains unclear which indices might be even more advantageous than others for predicting NAFLD, especially for Chinese subjects, especially under the present situation that the prevalence of overweight and obesity has increased a lot in China[28]. This study aimed to evaluate the performance of obesity-related indices, including Systolic Blood Pressure (SBP), Diastolic Blood Pressure (DBP), Fasting Blood Glucose (FBG), TC, TG, Low Density Lipoprotein (LDL), Waist Circumference (WC), A Body Shape Index (ABSI), Atherogenic Index of Plasma (AIP), Abdominal Volume Index (AVI), Body Adiposity Index (BAI), BMI, Body Roundness Index (BRI), Conicity Index (CI), TyG, Waist Hip Ratio (WHR) and Waist Height Ratio (WHtR) in identifying NAFLD in Chinese adults. Hopefully we will provide theoretical basis for utilizing anthropometric and metabolic indices to predict NAFLD in China.

Methods

Study population

We recruited participants in physical examination Center of Suzhou in southeast of China, during January 2020 to December 2020 cross-sectionally. Subjects included in this study were Chinese Han ethnicity with the age over 18 years old. A total of 1748 subjects were finally collected in the analysis after excluding those with alcohol abuse, other known causes of chronic liver disease and with missing or invalid data(Fig 1). The ethical committee of the Affiliated Suzhou Hospital of Nanjing Medical University approved this study (approval no. KL9011 study 12) and we have also got approval from all subjects who had agreed to participate into the present study.

Data collection

In the morning health examination was performed by expert medical staff. Health checkup and blood markers was measured as described previously by Xie et al[19]. In brief, weight and height were measured in light indoor clothing without shoes and heavy clothes, using a calibrated measuring apparatus. WC and Hip Circumference (HC) were measured as the horizontal circumference of the passing through the navel position and the most bulge at the hip, respectively. SBP and DBP were measured by sphygmomanometer. Metabolic markers, including TC, TG, LDL,

HDL and FBG were measured biochemically within 3 hours after peripheral blood drawn.

The obesity-related indices, including ABSI, AIP, AVI, BAI, BMI, BRI, CI, TyG, WHR, WHtR were calculated using these equations in Figure 2[20, 21, 25, 29].

Diagnoses of NAFLD was based on "Chinese Guideline on Diagnosis and Treatment of NAFLD (2006)" (diffuse hyperechoic liver relative to kidney, ultrasound beam attenuation, and weakening visualization of intrahepatic structures) by experienced radiologists with expertise in liver imaging. The NAFLD diagnosis met the two of the above three items. In addition, patients with no history of drinking or alcohol intake less than 40g in males or 20g in females per day over 5 years can be included in this study.[30]

Statistical analysis

The categorical variable was expressed as number (percentage). Continuous variables were expressed as median and two specific percentiles (P_{25} and P_{75}) for non-normal distributed data owing to . All participants were divided into NAFLD and non- NAFLD groups. The baseline variables (gender, age, anthropometric and metabolic indices) were compared using the Chi-square test and Rank tests appropriately.

SBP, DBP, FBG, TC, TG, LDL, WC, ABSI, AIP, AVI, BAI, BMI, BRI, CI, TyG, WHR and WHtR were divided into 4 quartiles according to their own changes respectively. The first quartile was used as a reference. Logistic analyses were performed to determine the associations between these anthropometric and metabolic indices and NAFLD with odds ratio (ORs) and 95% Confidence Intervals (CI).

Receiver Operating Characteristic (ROC) curves and Areas Under Curves (AUCs) were generated to compare the predictive ability of the various indices for identify NAFLD.

All statistical analyses were performed with the Statistical Package for the Sciences (SPSS, version 23.0). A value of $P<0.05$ in two-tailed test was considered significant.

Results

Characteristics of the Study Population

A total of 1748 subjects were included in our study, including 526 (30.09%) patients and 1222 (69.91%) controls. The mean age of patients and controls were 48.55±14.21 and 46.18±14.74

years ($P<0.01$). In these subjects, 464 (26.54%) males and 62 (3.55%) females had NAFLD. Table 1 compared the demographic characteristics, anthropometric and metabolic indices of individuals in groups. The percentage of NAFLD in males is higher than that in females, and the age of female subjects with NAFLD was significantly older than those without NAFLD. Subjects with NAFLD had higher SBP, DBP, FBG TC, TG, LDL, WC, ABSI, AIP, AVI, BAI, BMI, BRI, CI, TyG, WHR and WHtR both in males and females' groups, with significant differences (all $P<0.01$).

ORs for NAFLD Risk Across Quartiles of Each Index

Table 2 demonstrated that the analyzed parameters were significantly associated with NAFLD($P<0.01$). The ORs for NAFLD still increased across the quartiles of each index all in males and females after adjusting gender and age. Among the whole subjects, BMI showed the highest risk with NAFLD among all the indices, followed by WC and AVI.

ROC Curves and AUC for Indices in Identifying NAFLD

Table 3 showed that BMI had the greatest AUC with 0.841, while WC and AVI showed the same ability for predicting NAFLD in second (AUC = 0.836) among all the indices. The Youden index values of them were over 0.5.

In Figure 3, BMI and BRI had the same diagnostic ability for NAFLD (AUC = 0.812), followed by WHtR (AUC = 0.810) in males. In females, BRI also had the greatest predict ability (AUC = 0.849), and WHtR (AUC = 0.846) had the second greatest predict ability, followed by BMI (AUC = 0.844).

Figure 4 presented the ROC curves and AUCs of indices for NAFLD in age 20~, 40~ and 60~. Of all three ages, BMI and WC both showed predicting ability in the top three. AVI had the greatest AUC in age 20~ (AUC = 0.892) and age 40~ (AUC = 0.831), while TyG had higher AUC than AVI in age 60~ (AUC = 0.757). Besides, DBP, TC, LDL and ABSI in age 60~ had no significant predictive ability for subjects ($P>0.05$).

Discussion

This cross-sectional survey comprehensively evaluated the predictive ability and cutoff value of these obesity related anthropometric and metabolic indices to identify NAFLD and found BMI had the greatest AUC among all participants. Additionally, sex and age-specific indices for predicting

NAFLD existed.

Age and gender might be critical factors affecting the prevalence of NAFLD [3, 4, 11].The study found that men had a similar prevalence of NAFLD regardless of age, whereas it increased steadily with age in women. This is also in consistent with a previous finding, which reported that aging is a risk factor for NAFLD in Japanese women, independently from weight gain or influence of metabolic syndrome[31]. The increased prevalence of NAFLD with age for females might be associated with the alterations of sex hormones post-menopause. Visceral adiposity may be caused by loss of estrogen after menopause which may lead to extensive changes in the metabolic system. Generaaly, NAFLD is primarily considered asa male disease, however, the alteration in sex hormone levels, specifically reduced estrogens and increased androgens during and after menopause, might play important role in the emergence of NAFLD for female subjects[32, 33]. Investigators should also pay attention to NAFLD with increased age in Chinese females. Epidemiological studies propose a causative link between obesity and progressive liver disease in individuals, which is not only to the initial stages of the disease, but also to its severity [18, 34]. The pathophysiology and clinical studies have shown that the imbalance between lipid uptake and lipid disposal may cause oxidative stress and hepatocyte injury eventually[35]. Some studies thought that the visceral adiposity was the main adipose depot responsible for NAFLD and was associated with NAFLD in a dose-dependent manner in a cohort study[36]. WC, WHR, WHtR and BMI have been proved and used in many clinical trials as an indicator for the severity of fatty liver disease[21, 24]. ABSI considers the adjustment for height and WC in the calculation compared with BMI[37]. AVI is used to assess general volume, and it has been highly associated with dysfunction of glucose metabolism[38]. Increased AIP might be concordantly associated with the incidence of NAFLD [19, 20]. BAI is a better and easily applicable measure for determination of body fat compared with BMI, WHR, WHtR and WC in Turkish adults[39]. BRI was used to predict body fat, the percentage of visceral adipose tissue by Thomas et al.[40] TyG are often used to explore the relationship between insulin resistance and excessive visceral fat accumulation[41, 42]. Additionally, accumulating evidence strongly suggests that advanced blood lipids, blood pressure and blood sugar could also lead to more severe histological changes and poorer clinical outcomes[17, 24, 43]. Also insulin resistance can promote the progression of NAFLD to the more

severe state of liver endangerment like non-alcoholic steatohepatitis. This study further revealed that BMI and BRI had the relatively high association and diagnostic ability (0.812 in men and 0.849 in women) with NAFLD after considering the influence of gender, which is also supported by the research by Nima Motamed et al. who calculated AUC for BRI and WHtR (0.85 in men and 0.86 in women)[44]. The subtle differences between the two studies may be owing to difference between Chinese and Iranian. Findings (AVI had the greatest AUC in age 20~ and age 40~) is agreed with the study by Filippo Procino et al., who reported AVI had a low false negative rate and a higher percentage in identifying NAFLD, it should be mentioned that their research may be more helpful in NAFLD prediction for people in 20~59 age[45]. As we all know, diabetes is one of the strongest risk factors for NAFLD, the increasing prevalence of diabetes along with age especially in female subjects [15, 46] may explain the result that AVI had the greatest AUC in age 20~ and age 40~, while TyG had higher AUC than AVI in age 60~.

Strengths and limitations of the study

One of the biggest strength of this study is that almost all obesity-related anthropometric and metabolic indices were included in this study to be comprehensively evaluated in different gender and age. Although the association between these obesity related indices and NAFLD was analyzed separately in many articles, few articles have put them together to evaluate, especially in China. However, there are still some limitations. First, this is a cross-sectional study. Second, the data of other confounders, such as, smoking and drinking status and exercise, were not included in analysis because of the information default. Third, the ultrasonography diagnosis is a fast, reliable, reproducible and invasive method compared with liver biopsy, but it is unable to adequately determine the levels of steatosis and fibrosis.

Conclusion

This study found sex and age-specific indices for predicting NAFLD in Chinese subjects. For the whole population, BMI might be the best predictor for NAFLD, followed by WC and AVI. When stratified by sex, BRI and BMI both might be the best predictor for NAFLD in males, also BRI was suitable for predicting NAFLD in females. Considering age, AVI had the greatest AUC for those aged 20~ 60 years, while TyG had the higher predicted ability in 60~ years. Compared with

indices for all-age groups, sex and age-specific indices can provide more accurate assistance for clinical diagnosis and treatment. Besides, clues of disease cause can be found by comparing sex and age-specific indices

List of abbreviations

NAFLD: Non-alcoholic Fatty Liver Disease

SBP: Systolic Blood Pressure

DBP: Diastolic Blood Pressure

FBG: Fasting Blood Glucose

TC: Total Cholesterol

TG: Triglyceride

LDL: Low Density Lipoprotein

WC: Waist Circumference

ABSI: A Body Shape Index

AIP: Atherogenic Index of Plasma

AVI: Abdominal Volume Index

BAI: Body Adiposity Index

BMI: Body Mass Index

BRI: Body Roundness Index

CI: Conicity Index

TyG: Triglyceride Glucose

WHR: Waist–hip Ratio

WHtR: Waist-to-Height Ratio

OR: Odds Ratio

ROC: Receiver Operating Characteristic

AUC: Areas Under Curve

Ethics approval and consent to participate

The was approved by the ethical committee of the Affiliated Suzhou Hospital of Nanjing Medical

University (KL9011 study 12). Subjects agreed to participate into the present study and had provided a written informed consent.

Consent for publication

Not applicable

Availability of data and materials

The datasets generated and analysed during the current study are not publicly available due personal privacy but are available from the corresponding author on reasonable request.

Competing interests

The authors declare that they have no competing interests.

Funding

No funding.

Author Contributions

PYY and ZQ contributed to the study planning and design, ethics applications, organisational collaborations, data collection, analysis and interpretation, and writing of the manuscript. WY and CDL contributed to the study design, ethics applications, organisational collaborations, data collection, data interpretation and manuscript revision. XFF contributed to the study design, ethics applications, data interpretation and manuscript revision. All authors read and approved the final manuscript.

Acknowledgements

Not applicable.

References

1. Liu K, McCaughan GW: **Epidemiology and Etiologic Associations of Non-alcoholic Fatty Liver Disease and Associated HCC.** *Adv Exp Med Biol* 2018, **1061**:3-18.

2. Leoni S, Tovoli F, Napoli L, Serio I, Ferri S, Bolondi L: **Current guidelines for the management of non-alcoholic fatty liver disease: A systematic review with comparative analysis.** *World J Gastroenterol* 2018, **24:**3361-3373.
3. Araujo AR, Rosso N, Bedogni G, Tiribelli C, Bellentani S: **Global epidemiology of non-alcoholic fatty liver disease/non-alcoholic steatohepatitis: What we need in the future.** *Liver Int* 2018, **38 Suppl 1:**47-51.
4. Andronescu CI, Purcarea MR, Babes PA: **Nonalcoholic fatty liver disease: epidemiology, pathogenesis and therapeutic implications.** *J Med Life* 2018, **11:**20-23.
5. Perumpail BJ, Khan MA, Yoo ER, Cholankeril G, Kim D, Ahmed A: **Clinical epidemiology and disease burden of nonalcoholic fatty liver disease.** *World J Gastroenterol* 2017, **23:**8263-8276.
6. Sanyal AJ: **Past, present and future perspectives in nonalcoholic fatty liver disease.** *Nature Reviews Gastroenterology & Hepatology* 2019, **16:**377-386.
7. Marchisello S, Di Pino A, Scicali R, Urbano F, Piro S, Purrello F, Rabuazzo AM: **Pathophysiological, Molecular and Therapeutic Issues of Nonalcoholic Fatty Liver Disease: An Overview.** *Int J Mol Sci* 2019, **20**.
8. Farzanegi P, Dana A, Ebrahimpoor Z, Asadi M, Azarbayjani MA: **Mechanisms of beneficial effects of exercise training on non-alcoholic fatty liver disease (NAFLD): Roles of oxidative stress and inflammation.** *Eur J Sport Sci* 2019, **19:**994-1003.
9. Hernandez-Rodas MC, Valenzuela R, Videla LA: **Relevant Aspects of Nutritional and Dietary Interventions in Non-Alcoholic Fatty Liver Disease.** *Int J Mol Sci* 2015, **16:**25168-25198.
10. Valenzuela R, Videla LA: **The importance of the long-chain polyunsaturated fatty acid n-6/n-3 ratio in development of non-alcoholic fatty liver associated with obesity.** *Food Funct* 2011, **2:**644-648.
11. Wijarnpreecha K, Panjawatanan P, Aby E, Ahmed A, Kim D: **Nonalcoholic fatty liver disease in the over-60s: Impact of sarcopenia and obesity.** *Maturitas* 2019, **124:**48-54.
12. Agbim U, Carr RM, Pickett-Blakely O, Dagogo-Jack S: **Ethnic Disparities in Adiposity: Focus on Non-alcoholic Fatty Liver Disease, Visceral, and Generalized Obesity.** *Curr Obes Rep* 2019, **8:**243-254.
13. Mantovani A, Byrne CD, Bonora E, Targher G: **Nonalcoholic Fatty Liver Disease and Risk of Incident Type 2 Diabetes: A Meta-analysis.** *Diabetes Care* 2018, **41:**372-382.
14. Polyzos SA, Kountouras J, Mantzoros CS: **Adipose tissue, obesity and non-alcoholic fatty liver disease.** *Minerva Endocrinol* 2017, **42:**92-108.
15. Chao HW, Chao SW, Lin H, Ku HC, Cheng CF: **Homeostasis of Glucose and Lipid in Non-Alcoholic Fatty Liver Disease.** *Int J Mol Sci* 2019, **20**.
16. Targher G, Li Y, Wang J, Tang Y, Han X, Liu B, Hu H, Li X, Yang K, Yuan J, et al: **Bidirectional association between nonalcoholic fatty liver disease and type 2 diabetes in Chinese population: Evidence from the Dongfeng-Tongji cohort study.** *Plos One* 2017, **12:**e0174291.
17. Arrese M, Barrera F, Triantafilo N, Arab JP: **Concurrent nonalcoholic fatty liver disease and type 2 diabetes: diagnostic and therapeutic considerations.** *Expert Review of Gastroenterology & Hepatology* 2019, **13:**849-866.
18. Li L, Liu DW, Yan HY, Wang ZY, Zhao SH, Wang B: **Obesity is an independent risk factor**

for non-alcoholic fatty liver disease: evidence from a meta-analysis of 21 cohort studies. *Obesity Reviews An Official Journal of the International Association for the Study of Obesity* 2016, **17:**510.
19. Xie F, Zhou H, Wang Y: **Atherogenic index of plasma is a novel and strong predictor associated with fatty liver: a cross-sectional study in the Chinese Han population.** *Lipids Health Dis* 2019, **18:**170.
20. Wang Q, Zheng D, Liu J, Fang L, Li Q: **Atherogenic index of plasma is a novel predictor of non-alcoholic fatty liver disease in obese participants: a cross-sectional study.** *Lipids Health Dis* 2018, **17:**284.
21. VanWagner LB, Khan SS, Ning H, Siddique J, Lewis CE, Carr JJ, Vos MB, Speliotes E, Terrault NA, Rinella ME, et al: **Body mass index trajectories in young adulthood predict non-alcoholic fatty liver disease in middle age: The CARDIA cohort study.** *Liver Int* 2018, **38:**706-714.
22. Chen Z, Qin H, Qiu S, Chen G, Chen Y: **Correlation of triglyceride to high-density lipoprotein cholesterol ratio with nonalcoholic fatty liver disease among the non-obese Chinese population with normal blood lipid levels: a retrospective cohort research.** *Lipids in Health and Disease* 2019, **18**.
23. Rachakonda V, Wills R, DeLany JP, Kershaw EE, Behari J: **Differential Impact of Weight Loss on Nonalcoholic Fatty Liver Resolution in a North American Cohort with Obesity.** *Obesity (Silver Spring)* 2017, **25:**1360-1368.
24. Ren XY, Shi D, Ding J, Cheng ZY, Li HY, Li JS, Pu HQ, Yang AM, He CL, Zhang JP, et al: **Total cholesterol to high-density lipoprotein cholesterol ratio is a significant predictor of nonalcoholic fatty liver: Jinchang cohort study.** *Lipids Health Dis* 2019, **18:**47.
25. Li Y, Zheng R, Li J, Feng S, Wang L, Huang Z: **Association between triglyceride glucose-body mass index and non-alcoholic fatty liver disease in the non-obese Chinese population with normal blood lipid levels: a secondary analysis based on a prospective cohort study.** *Lipids Health Dis* 2020, **19:**229.
26. Guo W, Lu J, Qin P, Li X, Zhu W, Wu J, Xu N, Zhang Q: **The triglyceride-glucose index is associated with the severity of hepatic steatosis and the presence of liver fibrosis in non-alcoholic fatty liver disease: a cross-sectional study in Chinese adults.** *Lipids Health Dis* 2020, **19:**218.
27. Wang Y, Zeng Y, Lin C, Chen Z: **Hypertension and non-alcoholic fatty liver disease proven by transient elastography.** *Hepatology Research* 2016, **46:**1304-1310.
28. Pan XF, Wang L, Pan A: **Epidemiology and determinants of obesity in China.** *Lancet Diabetes Endocrinol* 2021, **9:**373-392.
29. Guerrero-Romero F, Rodrı guez-Mor á n M: **Abdominal volume index. an anthropometry-based index for estimation of obesity is strongly related to impaired glucose tolerance and type 2 diabetes mellitus.** *Archives of Medical Research* 2003.
30. Fan JG: **[An introduction of strategies for the management of nonalcoholic fatty liver disease (NAFLD) recommended by Asia Pacific Working Party on NAFLD].** *Zhonghua Gan Zang Bing Za Zhi* 2007, **15:**552-553.
31. Hamaguchi M, Kojima T, Ohbora A, Takeda N, Kato T: **Aging is a risk factor of**

nonalcoholic fatty liver disease in premenopausal women. *World Journal of Gastroenterology* 2012, **18:**237-243.

32. Hörist-Kollmann S, Strametz-Juranek J: **Female Dietary Patterns and the Pathogenesis of NAFLD.** *Gender and the Genome* 2018, **2:**49-55.
33. Cai M-J, Kong X-N, Zhao X-Y: **Influences of Gender and Age on the Prevalence and Complications of Nonalcoholic Fatty Liver Disease.** *Acta Academiae Medicinae Sinicae* 2017, **39:**499-505.
34. Mahli A, Hellerbrand C: **Alcohol and Obesity: A Dangerous Association for Fatty Liver Disease.** *Dig Dis* 2016, **34 Suppl 1:**32-39.
35. Cholankeril G, Wong RJ, Hu M, Perumpail RB, Yoo ER, Puri P, Younossi ZM, Harrison SA, Ahmed A: **Liver Transplantation for Nonalcoholic Steatohepatitis in the US: Temporal Trends and Outcomes.** *Digestive Diseases and Sciences* 2017, **62:**2915-2922.
36. Kim D, Chung GE, Kwak MS, Seo HB, Kang JH, Kim W, Kim YJ, Yoon JH, Lee HS, Kim CY: **Body Fat Distribution and Risk of Incident and Regressed Nonalcoholic Fatty Liver Disease.** *Clin Gastroenterol Hepatol* 2016, **14:**132-138 e134.
37. Christakoudi S, Tsilidis KK, Muller DC, Freisling H, Weiderpass E, Overvad K, Soderberg S, Haggstrom C, Pischon T, Dahm CC, et al: **A Body Shape Index (ABSI) achieves better mortality risk stratification than alternative indices of abdominal obesity: results from a large European cohort.** *Sci Rep* 2020, **10:**14541.
38. Quaye L, Owiredu W, Amidu N, Dapare PPM, Adams Y: **Comparative Abilities of Body Mass Index, Waist Circumference, Abdominal Volume Index, Body Adiposity Index, and Conicity Index as Predictive Screening Tools for Metabolic Syndrome among Apparently Healthy Ghanaian Adults.** *J Obes* 2019, **2019:**8143179.
39. Yeşil E, Köse B, Özdemir M: **Is Body Adiposity Index a Better and Easily Applicable Measure for Determination of Body Fat?** *Journal of the American College of Nutrition* 2020, **39:**700-705.
40. Thomas DM, Bredlau C, Bosy-Westphal A, Mueller M, Shen W, Gallagher D, Maeda Y, McDougall A, Peterson CM, Ravussin E, Heymsfield SB: **Relationships between body roundness with body fat and visceral adipose tissue emerging from a new geometrical model.** *Obesity (Silver Spring)* 2013, **21:**2264-2271.
41. Umano GR, Shabanova V, Pierpont B, Mata M, Nouws J, Trico D, Galderisi A, Santoro N, Caprio S: **A low visceral fat proportion, independent of total body fat mass, protects obese adolescent girls against fatty liver and glucose dysregulation: a longitudinal study.** *Int J Obes (Lond)* 2019, **43:**673-682.
42. Nirengi S, Fujibayashi M, Furuno S, Uchibe A, Kawase Y, Sukino S, Kawaguchi Y, Minato S, Kotani K, Sakane N: **Nonalcoholic Fatty Liver Disease in University Rugby Football Players.** *Front Endocrinol (Lausanne)* 2018, **9:**341.
43. Oikonomou D, Georgiopoulos G, Katsi V, Kourek C, Tsioufis C, Alexopoulou A, Koutli E, Tousoulis D: **Non-alcoholic fatty liver disease and hypertension: coprevalent or correlated?** *Eur J Gastroenterol Hepatol* 2018, **30:**979-985.
44. Motamed N, Rabiee B, Hemasi GR, Ajdarkosh H, Khonsari MR, Maadi M, Keyvani H, Zamani F: **Body Roundness Index and Waist-to-Height Ratio are Strongly Associated With Non-Alcoholic Fatty Liver Disease: A Population-Based Study.** *Hepat Mon* 2016, **16:**e39575.

45. Zheng RD, Chen ZR, Chen JN, Lu YH, Chen J: **Role of Body Mass Index, Waist-to-Height and Waist-to-Hip Ratio in Prediction of Nonalcoholic Fatty Liver Disease.** *Gastroenterol Res Pract* 2012, **2012:**362147.

46. Harsha Varma S, Tirupati S, Pradeep TVS, Sarathi V, Kumar D: **Insulin resistance and hyperandrogenemia independently predict nonalcoholic fatty liver disease in women with polycystic ovary syndrome.** *Diabetes Metab Syndr* 2019, **13:**1065-1069.

Table and figure legends

Table 1. Demographic, anthropometric, and metabolic characteristics of the study participants.

Table 2. ORs for FL stratified by quartiles of each index.

Table 3. AUC, Youden index, sensitivity, specificity and cut-off point of clinical parameters and obesity-related indices for predicting FL.

Figure 1. Comparison of the predictive value of FL-related parameters for diagnosis of FL among males and females.

Figure 2. Comparison of the predictive value of FL-related parameters for diagnosis of FL among age20~, 40~ and 60~.

NAFLD

ORIGINALITY REPORT

52%
SIMILARITY INDEX

PRIMARY SOURCES

1	Fangfei Xie, Yuyu Pei, Quan Zhou, Yun Wang, Renfang Han, Nimei Zeng, Kangyun Sun. "WHtR may be a Better and Stronger Indictor for Fatty Liver: A Population-Based Cross-sectional Comparison Study of Anthropometric and Metabolic Indices for Identification Fatty Liver in China", Research Square, 2021<br>Crossref Posted Content	1148 words — 32%
2	www.researchsquare.com<br>Internet	195 words — 5%
3	lipidworld.biomedcentral.com<br>Internet	92 words — 3%
4	Oluseyi Adegoke, Obianuju B. Ozoh, Ifedayo A. Odeniyi, Babawale T. Bello et al. "Prevalence of obesity and an interrogation of the correlation between anthropometric indices and blood pressures in urban Lagos, Nigeria", Scientific Reports, 2021<br>Crossref	33 words — 1%
5	I-Ting Lin, Mei-Yueh Lee, Chih-Wen Wang, Da-Wei Wu, Szu-Chia Chen. "Gender Differences in the Relationships among Metabolic Syndrome and Various Obesity-Related Indices with Nonalcoholic Fatty Liver Disease in a Taiwanese Population", International Journal of Environmental Research and Public Health, 2021<br>Crossref	31 words — 1%

6	Gerardo Luis Dimaguila, Frances Batchelor, Mark Merolli, Kathleen Gray. "'We are very individual': anticipated effects on stroke survivors of using their person-generated health data", BMJ Health & Care Informatics, 2020<br>Crossref	28 words — 1%
7	pubmed.ncbi.nlm.nih.gov<br>Internet	26 words — 1%
8	Helda Tutunchi, Fatemeh Naeini, Majid Mobasseri, Alireza Ostadrahimi. "Triglyceride glucose (TyG) index and the progression of liver fibrosis: A cross-sectional study", Clinical Nutrition ESPEN, 2021<br>Crossref	25 words — 1%
9	journals.plos.org<br>Internet	21 words — 1%
10	Dimitrios Oikonomou, Georgios Georgiopoulos, Vassiliki Katsi, Chris Kourek et al. "Non-alcoholic fatty liver disease and hypertension", European Journal of Gastroenterology & Hepatology, 2018<br>Crossref	19 words — 1%
11	academic.oup.com<br>Internet	17 words — < 1%
12	worldwidescience.org<br>Internet	17 words — < 1%
13	Haoyu Wang, Aihua Liu, Tong Zhao, Xun Gong et al. "Comparison of anthropometric indices for predicting the risk of metabolic syndrome and its components in Chinese adults: a prospective, longitudinal study", BMJ Open, 2017<br>Crossref	16 words — < 1%

14 res.mdpi.com
Internet
16 words — < 1%

15 link.springer.com
Internet
15 words — < 1%

16 Fangfei Xie, Hong Zhou, Yun Wang. "Atherogenic index of plasma is a novel and strong predictor associated with fatty liver: a cross-sectional study in the Chinese Han population", Lipids in Health and Disease, 2019
Crossref
14 words — < 1%

17 Nima Motamed, Behnam Rabiee, Gholam Reza Hemasi, Hossein Ajdarkosh et al. "Body Roundness Index and Waist-to-Height Ratio are Strongly Associated With Non-Alcoholic Fatty Liver Disease: A Population-Based Study", Hepatitis Monthly, 2016
Crossref
14 words — < 1%

18 pericles.pericles-prod.literatumonline.com
Internet
12 words — < 1%

19 K.-C. Sung, D.-C. Seo, S.-J. Lee, M.-Y. Lee, S.H. Wild, C.D. Byrne. "Non alcoholic fatty liver disease and risk of incident diabetes in subjects who are not obese", Nutrition, Metabolism and Cardiovascular Diseases, 2019
Crossref
11 words — < 1%

20 dagensdiabetes.se
Internet
11 words — < 1%

21 nutritionandmetabolism.biomedcentral.com
Internet
11 words — < 1%

22 www.semanticscholar.org
Internet
11 words — < 1%

23 Filippo Procino, Giovanni Misciagna, Nicola Veronese, Maria G. Caruso et al. "Reducing NAFLD-screening time: A comparative study of eight diagnostic methods offering an alternative to ultrasound scans", Liver International, 2018
Crossref
10 words — < 1%

24 Kun Lian, Yu - Nan Feng, Rong Li, Hao - Lin Liu, Peng Han, Lei Zhou, Cheng - Xiang Li, Qin Wang. "Middle - and high - molecular weight adiponectin levels in relation to nonalcoholic fatty liver disease", Journal of Clinical Laboratory Analysis, 2019
Crossref
10 words — < 1%

25 Youfa Wang, Li Zhao, Liwang Gao, An Pan, Hong Xue. "Health policy and public health implications of obesity in China", The Lancet Diabetes & Endocrinology, 2021
Crossref
9 words — < 1%

26 www.mdpi.com
Internet
9 words — < 1%

27 Bellan M, Menegatti M, Ferrari C, Carnevale Schianca GP, M. Pirisi. "Ultrasound-assessed visceral fat and associations with glucose homeostasis and cardiovascular risk in clinical practice", Nutrition, Metabolism and Cardiovascular Diseases, 2018
Crossref
8 words — < 1%

28 Helda Tutunchi, Maryam Saghafi-Asl, Mohammad-Javad Hosseinzadeh Attar, Mohammad Asghari-Jafarabadi, Alireza Ostadrahimi. "Food insecurity and lipid profile abnormalities are associated with an increased risk of non- alcoholic fatty liver disease (NAFLD): a case-control study in northwest of Iran", Research Square, 2020
Crossref Posted Content
8 words — < 1%

29 Jinjian Xu, Liqun Zhang, Qiong Wu, Yaohan Zhou, Ziqi Jin, Zhijian Li, Yimin Zhu. "Body roundness index is a superior indicator to associate with the cardio‐metabolic risk: evidence from a cross‐sectional study with 17,000 Eastern-China adults", BMC Cardiovascular Disorders, 2021 — 8 words — < 1%
Crossref

30 Tamoore Arshad, Pegah Golabi, James Paik, Alita Mishra, Zobair M. Younossi. "Prevalence of Nonalcoholic Fatty Liver Disease in the Female Population", Hepatology Communications, 2018 — 8 words — < 1%
Crossref

31 Jeffrey D. Browning, Lidia S. Szczepaniak, Robert Dobbins, Pamela Nuremberg et al. "Prevalence of hepatic steatosis in an urban population in the United States: Impact of ethnicity", Hepatology, 2004 — 7 words — < 1%
Crossref

EXCLUDE QUOTES	OFF	EXCLUDE MATCHES	OFF
EXCLUDE BIBLIOGRAPHY	ON		
